# Supplementary material for: Immunogenicity and Safety Profile of Two Adjuvanted-PD-L1-Based Vaccine Candidates in Mice, Rats, Rabbits, and Cynomolgus Monkeys
Source: Vaccines (Basel). 2025 Mar 11;13(3):296. doi: 10.3390/vaccines13030296 (PMC11946573; doi:10.3390/vaccines13030296)
Supplement: Supplementary file 1 [file vaccines-13-00296-s001.zip › vaccines-3401675-supplementary.pdf]

Supplementary Tables

Table S1. Qualitative parameters of BALB/c and C57BL/6 mice subcutaneously inoculated with PKPD-L1 mixed with VSSPs and aluminum phosphate.

	Experimental groups								
	BALB/c				C57BL/6				
	**1**	**2**	**3**	**4**	**5**	**6**	**7**	**8**	**Immunizations**
**Breathing**									
0-Normal, no tension or noise	0	0	0	0	0	0	0	0	**1st**
	0	0	0	0	0	0	0	0	**2nd**
1-Slightly increased or decreased	0	0	0	0	0	0	0	0	**3rd**
	0	0	0	0	0	0	0	0	**4th**
2-Fast or slow breathing	0	0			0	0			**5th**
	0	0			0	0			**6th**
12-Abdominal breathing with difficulty	0	0			0	0			**7th**
	0	0			0	0			**8th**
**Animal behavior**									
0-Normal	0	0	0	0	0	0	0	0	**1st**
	0	0	0	0	0	0	0	0	**2nd**
1-Minor changes with respect to normal behavior	0	0	0	0	0	0	0	0	**3rd**
	0	0	0	0	0	0	0	0	**4th**
2-Limited mobility and low alertness	0	0			0	0			**5th**
	0	0			0	0			**6th**
12-Vocalizations, self-mutilation, very restless or depressed	0	0			0	0			**7th**
	0	0			0	0			**8th**
**Appearance of the feces**									
	0	0	0	0	0	0	0	0	**1st**
0-Normal	0	0	0	0	0	0	0	0	**2nd**
	0	0	0	0	0	0	0	0	**3rd**
1-Soft	0	0	0	0	0	0	0	0	**4th**
	0	0			0	0			**5th**
2-Watery	0	0			0	0			**6th**
	0	0			0	0			**7th**
12-Persistent diarrhea for more than 24 hours	0	0			0	0			**8th**
**Appearance of the hair**									
0-Normal	0	0	0	0	0	0	0	0	**1st**
	0	0	0	0	0	0	0	0	**2nd**
1-Lack of grooming (shabby hair)	0	0	0	0	0	0	0	0	**3rd**
	0	0	0	0	0	0	0	0	**4th**
2-Shabby hair and/or ocular/nasal secretions	0	0			0	0			**5th**
	0	0			0	0			**6th**
3-Very bristly hair, abnormal posture	0	0			0	0			**7th**
	0	0			0	0			**8th**

Note: The animals belonging to groups 1 and 5 were inoculated with 200 µg of PKPD-L1 mixed with 100 µg VSSPs. The mice to groups 3 and 7 were inoculated with 200 µg of PKPD-L1 mixed with 0.7 mg of aluminum phosphate. The mice of the second and sixth groups received saline solution mixed with 100 µg VSSPs (vehicle control group). The mice of the fourth and eighth groups received saline solution mixed with 0.7 mg aluminum phosphate (vehicle control group).

Table S2. Hematological and serum biochemical parameters evaluated in rabbits and monkeys.

**Rabbits**	White blood cell count, red blood cells, hemoglobin, hematocrit, mean corpuscular volume, mean corpuscular hemoglobin, mean corpuscular hemoglobin concentration, platelets, neutrophils, lymphocytes, monocytes, alanine aminotransferase, aspartate aminotransferase, creatinine, total proteins, total bilirubin, albumin.
**Non-human primates**	White blood cell count, red blood cells, hemoglobin, hematocrit, mean corpuscular volume, mean corpuscular hemoglobin, mean corpuscular hemoglobin concentration, platelets, alanine aminotransferase, aspartate aminotransferase, creatinine, alkaline phosphatase, glucose, total bilirubin, direct bilirubin, triglycerides, phosphorus, urea, gama-glutamyl transferase.

Supplementary Figures

Supplementary Figure 1.

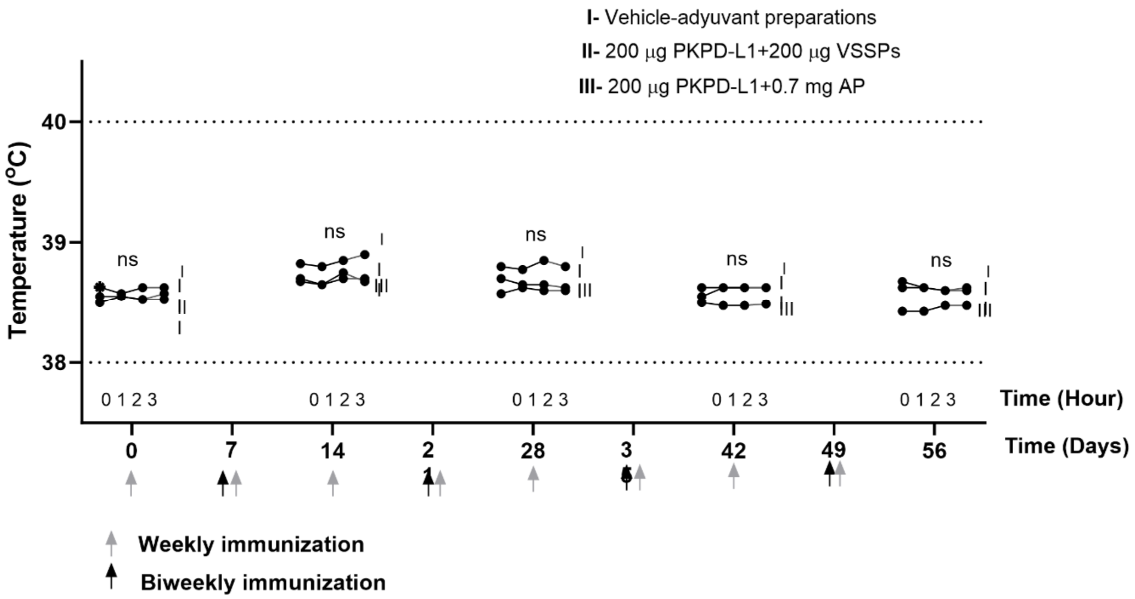

Figure S1. Time course of temperature in rabbits immunized with PKPD-L1. Each symbol indicates the average temperature of the rabbits (n=4) according to the time and day of the immunization schedule. Groups: (I) Vehicle-adjuvant preparations; (II) 200 μg PKPD-L1+200 μg VSSPs; (III) 200 μg PKPD-L1+0.7 mg AP. No differences were detected between each immunization group ($p > 0.05$, One-way ANOVA, Tukey post-test).

Supplementary Figure 2.

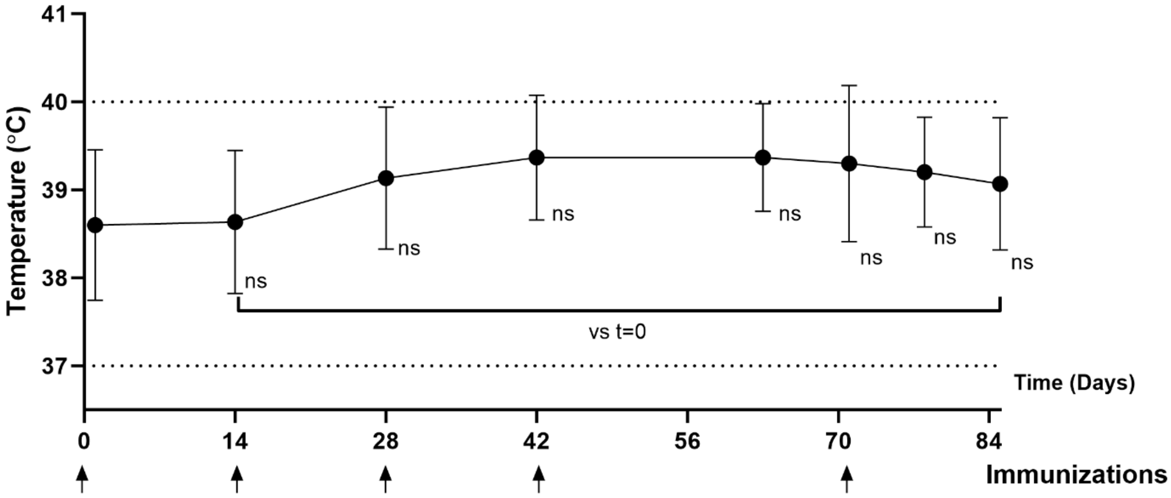

Figure S2. Time course of temperature in NHPs immunized with PKPD-L1. Each symbol indicates the mean and the standard deviation of the temperature of the NHPs (n=3) according to the day of the immunization schedule. Statistical significance is represented in the graph as ns $p \geq 0.05$; * $p<0.05$; ** $p<0.01$; *** $p<0.001$; **** $p<0.0001$ according to the paired Wilcoxon test (ns: not significant).

Supplementary Files

Supplementary File S1. Procedure of ELISA protocols

ELISA for detecting PD-L1 specific IgG human antibodies

Plates (Costar, 3590) were coated overnight at 4˚C with 1 μg/wells of hPD-L1-His diluted in PBS (pH 7.4). After three washes with 0.05% Tween 20 in PBS, 5% skim milk in PBS was added to the plates and incubated for 1 h at 37˚C, followed by new washes. Diluted sera (two-fold serial dilutions from 1:500 to 1:32000) from the experimental immunization groups were added to wells and incubated for 1 h at 37˚C. Wells were then washed and incubated with a 1:7500 dilution of sheep anti-mouse IgG HRP-conjugated antibody (for mice), a 1:10000 dilution of HRP-conjugated goat anti-rat IgG polyclonal (for rats), a 1:30000 dilution of HRP-conjugated goat anti-rabbit IgG polyclonal antibody (for rabbits) or a 1:10000 dilution of HRP-conjugated goat anti-human IgG polyclonal antibody (for non-human primates). After incubation for 1 h at 37°C, plates were washed again and incubated with TMB as substrate-chromogen solution for 10 minutes. After 15 minutes the reaction was stopped by adding 50 μl/well of 2N sulphuric acid solution. The absorbance was read at 450 nm in a BioRad microtiter plate reader.

Competitive ELISA for measuring serum-mediated inhibition of PD-1/PD-L1 interaction

Competitive ELISA has been previously described in details by Morera et al. [1]. Briefly, plates (Costar, 3590) were coated overnight at 4˚C with 10 ng/wells of human PD-1/Fc chimera diluted in PBS (pH 7.4). Equal volumes of sera from immunized mice at a 1/25 dilution were mixed with 50 ng/wells of human PD-L1/Fc chimera in 0.4% BSA in PBS (dilution buffer) and were incubated for 2 h at 37˚C to allow the formation of immune complexes. After immune complexes formation, free PD-L1 can bind to coated PD-1 and the binding PD-1/PD-L1 was detected with a biotinylated anti-human PD-L1 antibody, followed by a streptavidin-peroxidase conjugate. The maximum binding of PD-L1 was recorded in absence of any animal serum and a commercially available anti-human PD-L1 monoclonal antibody was used as an inhibition positive control. A similar procedure was conducted to evaluate the sera effects on CD80/PD-L1 interaction.

The percent of inhibition activity is represented by 100% – [(Absorbance 450 nm of serum sample /Absorbance 450 nm maximum binding of PD-L1) × 100].

Supplementary File S2. Mice welfare assessment

The qualitative evaluation was performed using numerically defined categories. The changes in breathing were evaluated as 0, normal breathing, no tension or noise; 1, breathing slightly increased or decreased; 2, fast or slow breathing and 12, abdominal breathing with difficulty. The changes in animal behavior were assessed according to 0, normal; 1, minor changes concerning normal behavior; 2, limited mobility and low alertness, isolated from other animals; and 12, vocalizations, self-mutilation, very restless or depressed. The appearance of the feces was categorized according to consistency in 0, normal; 1, soft; 2, watery; and 12, persistent diarrhea for more than 24 h. The appearance of the hair was assessed as 0, normal; 1, lack of grooming (shabby hair); 2, shabby hair and/or ocular/nasal secretions and 3, very bristly hair, abnormal posture. When the total score reached 12 or more points, the animal was euthanized.

[1] Y. Morera-Díaz, C. Canaán-Haden, J. Sánchez-Ramírez, M. Bequet-Romero, I. Gonzalez-Moya, R. Martínez, V. Falcón, D. Palenzuela, M. Ayala-Ávila, J.V. Gavilondo, Active immunization with a structurally aggregated PD-L1 antigen breaks T and B immune tolerance in non-human primates and exhibits in vivo anti-tumoral effects in immunocompetent mouse tumor models, Cancer Letters, 561 (2023) 216156.
